# The role of poetry and prose in medical education: the pen as mighty as the scalpel?

**DOI:** 10.1007/s40037-012-0008-1

**Published:** 2012-03-13

**Authors:** Frank J. Wolters, Marjo Wijnen-Meijer

**Affiliations:** 1 Department of Neurology, University Medical Centre Utrecht, PO Box 85500, 3508 GA Utrecht, the Netherlands; 2 Centre for Research and Development of Education, University Medical Centre Utrecht, HB 4.01A, PO Box 85500, 3508 GA Utrecht, the Netherlands

**Keywords:** Poetry, Prose, Literature, Medical education

## Abstract

The current medical students’ training on communication skills does not completely fulfil its purpose, since it often lacks attention to the various aspects essential for developing an empathic capacity. Besides having an active component in empathy, cognitive and affective aspects are of equal importance. Integration of arts and humanities courses into the curriculum might bridge this gap. Empathy results mainly from recognition and acknowledgement of a wide spectrum of emotions in patients, their relatives and doctors themselves. Artistic forms of reflection can promote insight into these emotions, complementary to the current teachings which focus on the active component of empathy. Based on several psychological and didactic views, as well as multicentre experience mainly from the United Kingdom and United States, poetry and prose can contribute to sustained development of empathy in medical students.

## Introduction on the origin of understanding

‘The art of painting reflects silent poetry. Painting is a form of poetry to be seen, rather than felt, and poetry is painting to be felt, rather than be seen.’ Leonardo da Vinci’s words mark the commitment between visual arts and literature, including poetry. Indeed, these artistic expressions, at first sight so diverse, have many similarities the greatest of which probably lies in the affective area. Art mirrors emotion. Of all the types of decorative artwork, painting has been a particularly popular form of art for use in medical education. In *arts and humanities* courses, mainly established in the United Kingdom and the United States, masters of the old such as Monet, Rembrandt and Picasso are posthumously offered the chance to contribute to young doctors’ training. Studies have shown that these courses improve both observational capacities as well as awareness and acknowledgement of emotional experiences amongst patients [1, 2]. The latter contributes to improved doctor–patient relationships, defined as one of the seven pillars of the* medical expert* in the CanMEDS framework in 1997 [3].

In recent decades empathy has become a centre of attention in medical education. However, universities lack consensus about the ideal way of teaching this aspect of medicine [4]. If optimal training in medicine is to be achieved then it is crucial that we look for educational strategies aimed at the multiple elements empathy consists of [4–6]. This includes a cognitive (understanding one another’s thoughts), affective (emotions towards one another), and an active component (confirmation in being heard and understood) [7]. All too often medical schools focus on the active component of empathy, overlooking the cognitive and affective aspects. Consequently, the professional attitude these schools strive for during the preclinical phase lacks a full-fledged foundation. As a result, the acquired empathic skills are susceptible to decline by harsh everyday reality during rotations [8, 9]. When medical students encounter uninspired physicians or suffer from a lack of time to spend at the patient’s bedside, this results in disillusion, cynicism, and even regression of previously acquired communication skills: [8, 9] a mismatch between the formal and hidden curriculum calling for change.

In order to train empathic young physicians it is essential to anatomize the concept of empathic attitude. What is understanding? And what do we mean by empathy as the ability to understand emotions? Understanding originates from fitting a certain event in a pattern of comparable events [5]. This happens in sociology, where one strives to understand human interaction by, for example, economic, religious and psychological patterns; the role pattern arises [5]. This approach is quite comparable with the way doctors identify diseases by clusters of symptoms, which we call* illness scripts* [10]. In his bestseller* How doctors think* Jerome Groopman issues a warning that this strategy can result in failure to see the whole picture [11] and how this reasoning all too often leads to medical malpractise [11]. In addition, the role pattern overlooks the uniqueness of the individual patient, at a time that we cherish a bespoke approach to medical care.

Consequently, medical educators carry the responsibility of delivering more ‘complete’ young doctors. Meites et al. defined a threefold backbone crucial in achieving this ambition. They address three key questions; how patients experience their illness and their doctors, and thirdly how illness alters one’s emotional landscape and consciousness [12]. Thus, on the way to becoming a doctor, much evolves around medical students’ relationships with patients and their relatives. Medical students are quite aware of this development and its importance, judging by poetry produced by third-year students at the University of California [13]. Asked to reflect upon a (thorough) clinical experience by self-chosen (creative) means, they wrote 171 poems during their internal medicine and paediatrics rotations. Over half of these poems had the aforementioned relationships as their primary theme (Table 1). In addition, 20% of the writings discussed the role of the supervising doctor. Unfortunately, these were not merely positive reflections [13]. Aiming for inventive improvements, the question raises whether literary activity can positively influence Meites’ landmarks.

**Table 1:** Themes in poetry as depicted by medical students, subtracted from Shapiro et al. [13]

Theme of the poem	Number of poems (%)
Student–patient relation	60 (35.1)
Student–patient’s family relation	35 (20.5)
Guidance and mentor observation	32 (18.7)
Other (death, stress, spirituality, etc.)	44 (25.7)
	171 (100)

In this article we explore the complementary value of literary activity in medical education, concerning the acquirement of empathic skills, in which the three different elements of empathy are of vital importance. In addition, we question how we can integrate these insights into medical students’ training programmes.

## Healing words

In assessing the additional value of poetry and prose as a didactic instrument, an important question is which of the earlier mentioned aspects of empathy, namely the cognitive, affective and active components, do we recall in poetry and prose? The answer to this question lies enclosed in one of the key objectives in medical education: regarding every patient as a unique human being. By focussing on individual experiences, literary experiences may contribute to the accomplishment of this goal. Professor in Moral Philosophy, R. S. Downie, wrote: ‘*The humanities […] are concerned with the particularity of situations and with their meaning, and that concern is the way to whole person understanding*’ [5]. This uniqueness and meaning of certain moments are exactly what we believe to be of importance in understanding emotions and adequate reflection. According to Downie, poetry, like novels, plays and movies, has the ability to greatly refine intuitive understanding in medical students and young doctors [5]. Downie refers to intuitive understanding, as opposed to the rational understanding from exact and social science. It is due to its individual approach that we cannot readily measure the empathic learning experience in terms of rational numbers [5]. Instead, we have to rely on our own (and our interlocutor’s) perception and understanding of the situation (to be tested by follow-up questions)—the cognitive component in empathy. Thus, the way we learn from exploring literature as an art is mainly by being able to imagine and subsequently identify a large variety of subjective experiences and emotions. Literary activity is thus a tool, extending our insights into patients’ behaviour and encouraging continuous reflection on our work by raising (moral) questions [5]. So, we have sufficient reason to believe literary activity contributes to increased cognitive empathic traits. Therefore, the incorporation of poetry and prose into the medical curriculum makes a profound contribution to medical education, in addition to existing teaching methods focussing around active empathy.

## A curricular stanza

In order to proceed we need to determine how to convert this reasoning into practice. To do this, we will examine several initiatives to promote literary activities in medical curricula around the world. As it turns out, most of the reported initiatives in incorporating literature courses are set in motion with a pilot group of volunteers, or as an elective course within the regular curriculum [12, 14, 15]. Results from several institutions show that poetry carries additional value in developing communicative skills and increasing detection and understanding of (one’s own and others’) emotions [15, 16]. Feedback by both participating students and tutors is overwhelmingly optimistic [15, 16]. One institution reports data from long-term programmes, extending beyond 5 years of implementation. During this period the poetry and prose courses at the University of California have developed from an elective course into a full-fledged part of the curriculum [17]. Similar tendencies are reported from the United Kingdom [14].

An important consideration when opting for the integration of literature courses into the curriculum is the choice between an elective course or an obligatory poetry programme. This choice may greatly influence its impact on the educational experience. It is likely that the additional value of the course will be higher for intrinsically motivated students. Unfortunately, poetry and prose generate only minor interest in the lives of students and doctors these days. This was already illustrated in the Lancet in 1995 by the London professor I. C. McManus, who stated, among other things, that:* ‘Concerns that medicine and perhaps doctors are becoming dehumanised recur throughout medical and non-medical published work*’ [18]. Consequently, there may be few applications for elective courses, while obliged integration in the education programme involves all medical students. The latter approach therefore seems preferable, since benefits from literary courses are indisputable, if implemented well. Wide implementation, reaching all medical students, can be achieved by using poetry for instance in reflection on clinical rotations [19], or in analyzing the emotional impact of early anatomy courses involving human corpses [20]. A ‘*Californian*’ approach involving elective courses as well as poetry intertwined throughout the curriculum seems advantageous, since it provides a way for all students to get acquainted with the topic, offers further challenge for those interested in mastering the subject, and as an initial concept has the ability to develop and expand over time [17]. Furthermore, a balanced course including, for example, poetry and prose, illustrative art and the history of medicine may attract the increased attention and efforts of students.

In whatever form of education, an important task is entrusted to the tutor. He or she carries the challenging responsibility of guiding the group, making the most of the students’ efforts in a dynamic group process. The tutor should therefore have insight into group dynamics and skills required to guide the workgroup, rather than be an expert in literary matters himself [16, 17]. Students can learn greatly from each other’s experiences and subsequent valuable discussions. Additionally, involving poets or other local artists in this process, as well as arranging encounters with alpha faculties, may increase the yields of the programme [16, 17]. Finally, implementation of poetry into education does not necessarily require extensive efforts. This is illustrated by the upcoming 55-word story. Exactly 55 words of poetry or prose may be used to express an experience or event. The conciseness —not to be mistaken for simplicity—stimulates the impact on both writer and reader. Submissions were published for example in the *Journal of the American Medical Association* (JAMA) and *Family Medicine*. Table 2 shows a brief guide towards writing such a 55-word story, the implementation of which requires solely a teacher’s inventiveness [21, 22].

**Table 2:** How to write a 55-word story [21, 30]

1	Think of a compelling story based on your experience (as clinician, patient, other?)
2	Write down everything you can think of
3	Don’t edit, just write (phrases, words, key chunks of memory)
4	Put it away (optional and can be done at any time between #2 and finishing)
5	Read over your writing and begin to clarify the idea or storyline that you want to convey.
6	Begin editing, sometimes ruthlessly
7	Share your work with others for reactions and feedback
8	Keep editing until you get 55 words. Use your word counter, and also double check manually.
	a. The title doesn’t contribute to the word count but shouldn’t be more than seven words
	b. Contractions count as single words
	c. Eliminating articles (the, a, an) can help with word count
9	If you cannot cut enough words, you probably have material that either would lend itself to a longer essay or become multiple 55-word stories
10	Given the brevity, formatting can make a big difference. Experiment with line length, indentations, hanging indents, and other use of white space

## A writer’s legacy

Once it has been decided to make literary activities an intrinsic part of medical education, art can be explored by either reflecting on existing artwork or creating one’s own poetry or prose. Both have the ability to incite empathy by stimulating mainly cognitive and affective growth. However, the origin of a poem largely determines its emotional weight and impact. Since the empathic evolvement centres around each and every student, reflection may well start at the students’ pen. However, it might be just as useful to reflect on patients’ poetry, experiences by patients’ relatives, or a physician’s prosaic point of view. Several patients and patient associations have published poetry and a notable example of medical students’ work can be found on the interactive website issuing* arts and humanities* projects by Bristol students [14, 21, 23].

Either way, efforts should be focussed around the personal feelings experienced while reading a poem. This pleads for a certain freedom of choice in selecting appropriate scripts. New Zealand ethicist* Neil Pickering clarifies: ‘Reading a poem involves personal engagement and is an unpredictable business. We can’t know in advance what will come out of it. And if we try to specify what should or must come out of it, we are standing in the way of reading the poem*’ [24]. Poetry should be studied for the poetry itself, avoiding a set framework, and assignments should therefore be as free as possible. Consequently, poetry and prose are frequently chosen expressions among the different art forms, such as music and expressive arts [13]. This allows students to stick to their personal preferences, encouraging participants to share their thoughts and concurrently giving them the incentive to make use of a certain art form as a means of reflection throughout further life and career. Furthermore, creativity is enhanced by assessment without grading. After all, the process of reflection and understanding is of greater importance than the sheer artistic quality of a contribution.

## Writers from around the world, unite

Up until now the vast majority of publications concerning* arts and humanities* courses in medical curricula originate from a handful of leading nations. In order to share experience and spread knowledge about* arts and humanities* courses extensive evaluation and international comparison and extrapolation are required. Although it is believed by some that the success of poetry cannot be measured by numbers [16, 24] a breakthrough in international implementation may well depend on the ability to clearly compare the results from various reports. Therefore, future studies may fare well by the use of universal scorecard indicators. Concerning empathic qualities, two methods of measurement stand out with reference to reliability and validity: the Empathy Construct Rating Scale (ECRS) [25] and the Balanced Emotional Empathy Scale (BEES) [26]. The ECRS consists of 20 items with a six-point scale, surveying self-consideration on traits such as listening, paraphrasing and emotional reflection. The BEES comprises 30 items, regarding susceptibility for someone else’s feelings on a nine-point scale. Use of both scales suggests that the ECRS is more suitable to assess cognitive and active aspects of empathy, whereas the BEES tends to centre around the affective facet [15]. Although the discriminative value of both tests remains to be fully elucidated, these findings point to the significant difference between various aspects of empathy.

## Conclusion

The role of literary activities in* arts and humanities* programmes is increasing. Poetry is applied by active (writing) and passive (analyzing) means, with both means being able to contribute to the relationships relevant to medical students, i.e. those with patients, patients’ families, and future colleagues. This training focuses mainly on the cognitive aspect of empathy, as a necessary supplement to the current teaching of clinical skills focussed around the active empathy component. Integrating poetry into the medical curricula, applied in either regular or elective courses, is well received by both students and tutors. Available literature suggests there should be freedom of choice in topic and expression, without summative evaluation, in order to maximally exploit the educational potential of poetry and prose.

Promising reports are coming in from around the globe, carrying many positive experiences with literary activity as an educational tool. However, despite increasing interest,* arts and humanities* courses remain relatively rare. Additional data is needed to tilt the balance in favour of broadened integration of poetry into the medical curriculum. Good-quality longitudinal approaches amongst representative student populations should become the first priority. Although this reflection focuses on medical students’ education, poetry has been a welcome guest in general practitioners’ training [27], as well as for residents [28, 29]. On various levels reflection encompasses empathic evolvement, with poetry as a powerful ally. As the renowned Chekhov once wrote: ‘Do you take medicine as your lawful wife, and literature as your mistress?’ [30].

## Essentials

- Empathy consists of multiple elements, partly overlooked in current medical education approaches.
- The use of literature in medical education can increase empathic development in young physicians by encouraging reflection and understanding.
- Poetry and prose can be integrated into medical education in numerous ways, from electives to full-fledged courses.
- Writing and reading of literature stimulates empathic development from different perspectives.
- Global incorporation of literature in medical education may be facilitated by well-designed international comparative studies, using standards such as empathy rating scales.
